# Prevalence and correlates of chronic diseases in an elderly population: A community-based survey in Haikou

**DOI:** 10.1371/journal.pone.0199006

**Published:** 2018-06-14

**Authors:** Chanjuan Zhao, Liping Wong, Qing Zhu, Hao Yang

**Affiliations:** 1 College of Public Health, Hainan Medical University, Haikou, Hainan Province, China; 2 Department of Social and Preventive Medicine, Faculty of Medicine, University of Malaya, Kuala Lumpur, Malaysia; 3 Department of Non-communicable Disease Control and Prevention, Haikou Center for Disease Control and Prevention, Haikou, Hainan Province, China; Istituto Di Ricerche Farmacologiche Mario Negri, ITALY

## Abstract

**Background:**

The escalating problem of multiple chronic conditions among older adults in China draws public health attention due to increasing proportion of the elderly population. This study sought to assess the prevalence of and factors associated with four chronic diseases in older adults in Haikou, the capital city of Hainan Province, China.

**Method:**

In this cross-sectional study, 9432 community-dwelling elderly people aged 60 years and older living in rural or urban areas in Haikou were investigated. The interviews collected self-reported information on the presence of four major chronic diseases, as well as socio-demographic characteristics, lifestyle factors and self-reported height and weight.

**Findings:**

Overall, 31.7% (2961/9344) reported at least one of the four chronic diseases. The prevalence of hypertension, diabetes mellitus, COPD, and stroke was 26.0% (2449/9407), 8.0% (749/9371), 1.0% (95/9360), and 1.9% (175/9382), respectively. Common correlates of the four major chronic diseases were older age, being engaged in intellectual work, currently being a smoker and obesity. Gender, locality of residence, and alcohol consumptions were also found to be associated to some of the chronic conditions.

**Conclusion:**

This finding indicates that multiple chronic conditions among elderly people in Haikou are prevalent and warrant special attention to reduce diseases burden and align health care services to cater the holistic elderly patients’ need.

## Introduction

The ageing population is a worldwide issue that will result in increased medical expenditure and workforce shortages in the elderly care sector in addition to other negative consequences. A recent overview indicated that the proportion of people aged 60 years and over is expected to double between 2007 and 2050, reaching 2 billion worldwide by 2050 [1]. As the world's largest developing country, China is also heading for an elderly population crisis, China’s elderly population expected to grow to 25% by 2030. China’s rapid aging process is not only result in the future rates of economic growth, but most importantly it may poses great challenge in meeting the financial and social service burdens of caring for the elderly. As such, one of China’s newest challenges is adapting to its aging population. With increase aging population, couple with increase life expectancy the leading cause of death may change from infections to chronic non communicably diseases [2]. It has been reported that major chronic illnesses that burden aging population in high-income countries were namely hypertension, high cholesterol, arthritis, diabetes, heart disease, cancer, and dementia [2]. The stress of the long-term care of aging population has now become a particular interest in our China.

In China, there is growing evidence that chronic diseases are the primary health issues in the elderly populations. Conditions such as hypertension, diabetes mellitus, stroke, cardiovascular disease, and cancer, are the leading causes of death in China, accounting for 86.6% of all deaths in 2012 [3]. Previous literature indicated that two large scale survey on prevalence of chronic conditions have been conducted in China, namely CNNHS (China National Nutrition and Health Survey) and Chronic Disease Risk Factor Surveillance in China [4, 5]. Nonetheless, the sample are general population and lack of representative of older adults. Most recently the SAGE (Study of global AGEing and adult health)- China Wave 1 cross-sectional study examined the prevalence of common chronic illness among the elderly in China. The study found half of elderly in China reported having at least one chronic illness and near one-fifth reported at least two chronic illnesses. However, the SAGE-China Wave 1 study recruited participants from eight provinces in China which did not include Hainan province. Haikou, the capital city of Hainan Province, is located in the north of Hainan island; it has a typical subtropical climate, abundant marine resources, and the most centenarians in China. According to a new census report, there were 157.4 thousand people 65 years and older in Haikou at the end of 2014, representing up to 7.15% of its total population. Despite the profound effects of aging nation on health and economic burden, chronic diseases among elderly population in Haikou remains understudied.

Exploring contributing factors of chronic diseases is extremely important in designing intervention and informs policy decision. Previous studies have implied that genetic factors may contribute to the lifespan of elderly Chinese people [5, 6, 7]. Recent studies have confirmed that an increased body weight, an unhealthy diet and a sedentary lifestyle can pose risks for chronic diseases [8, 9], and these risk factors show disparities by region as well as by socio-economic status [10]. Other behavioural factors such as excessive alcohol consumption and smoking were also found to be associated to chronic illness in elderly men [11]. Identifying the demographic as well as other modifiable risk factors contribute to the chronic diseases disparities in the elderly is a necessary step in developing appropriate interventions to eliminate or reducing the prevalence of chronic diseases.

In the SAGE-China Wave 1 study, little was mention about the behavioural factors related to chronic diseases in the Chinese elderly population [4]. Further, the prevalence of chronic diseases and related factors in the elderly population in Haikou has not been determined. Therefore, the aim of this study was to assess the prevalence of four chronic diseases (namely hypertension, diabetes mellitus, chronic obstructive pulmonary disease [COPD], and stroke) based on self-report and the associated demographic and behavioural factors in the older adult population in Haikou.

## Methods

### Study settings and data collection

This research was conducted within the framework of the Haikou Communities Health Assessment, a community-based cross-sectional study. The survey was conducted from November 2015 to January 2016 and involved a sample of 9432 community-dwelling elderly residents aged 60 years and older (4721 males and 4711 females). The sample was randomly selected from four administrative districts in Haikou using two-stage cluster sampling to control sampling error. In the first stage, all 43 communities and towns served by the 116 sites of the community health service centre were recruited in the sample frame. One to two neighbourhoods or villages were then selected from each community or town by using a random number generated according to population size. In the second stage, all permanent residents aged 60 years and over residing in the selected sample neighbourhoods or villages were visited and assessed.

Information was collected through face-to-face interviews with the respondents or with a household member for those who were reading disabled. Self-reported of four chronic diseases queried in this study were hypertension, diabetes mellitus, COPD, and stroke. Respondents’ chronic diseases statuses were obtained by asking “Have you ever been diagnosed with the following conditions?”

The questionnaire also assessed socio-demographic characteristics: gender, age (categorized into 60–69 years, 70–79 years and ≥80 years), household monthly income (≤1500 yuan [CNY], 1501–6000 yuan, and ≥6001 yuan), education (primary school or below, high school, technical school and university), occupation (intellectual worker, manual worker, and retiree). Finally, health behavioural factors, i.e., smoking (never, past smoker and current smoker) and alcohol intake (yes vs. no), and self-reported height and weight were also obtained.

This study was approved by the Hainan Medical University Research Ethics Committee (086 0898–66893600). The eligible elderly who agreed to participate in the study signed an informed consent form.

### Measurements

Standing height was measured using a stadiometer fixed to the wall and recorded to the nearest 0.1cm. Body weight was measured to the nearest 0.1kg using an electronic scale. Body Mass Index (BMI) was calculated as weight (kg) divided by height squared (m^2^).

### Statistical analysis

Sample estimates of self-reported chronic diseases (hypertension, diabetes mellitus, COPD, and stroke) stratified by socio-demographic characteristics and behavioural factors are reported, including absolute values, their respective percentages of the total study sample, and 95% confidence intervals (95%CIs). Binary logistic regression was used to evaluate the associations of self-reported chronic diseases with the suspected risk factors. Odds ratios (ORs; for example, OR of hypertension: “hypertension: yes” vs. “hypertension: no”) and their respective 95%CIs were calculated. Participants with missing data on BMI were excluded from regression model. IBM SPSS Statistics (version 20.0) was used for all analyses.

## Results

Overall, 31.7% (2961/9344) reported at least one of the four chronic diseases. The specific prevalence of hypertension, diabetes mellitus, COPD, and stroke was 26.0% (2449/9407), 8.0% (749/9371), 1.0% (95/9360), and 1.9% (175/9382), respectively (Table 1). By demographic characteristics, the prevalence of hypertension and diabetes mellitus did not differ between male and female participants, while the prevalence of both COPD and stroke was higher in men than in women (COPD 1.6% [male] vs. 0.5% [female]; stroke 2.3% [male] vs. 1.5% [female]). Participants who were aged 60–69 years had the lowest prevalence of the four chronic conditions (hypertension 20.5%, diabetes mellitus 7.1%, COPD 0.8%, and stroke 1.3%), whereas individuals aged 80 years and older exhibited the highest prevalence of hypertension (34.1%), COPD (2.0%) and stroke (2.9%,). Elderly individuals residing in urban areas had a significantly higher prevalence of hypertension and diabetes mellitus than the rural elderly (hypertension 31.2% [urban] vs. 19.7% [rural]; diabetes mellitus 11.0% [urban] vs. 4.3% [rural]) but a slightly lower prevalence of COPD and stroke than their rural counterparts (COPD 0.6% [urban] vs. 1.5% [rural]; stroke 1.5% [urban] vs. 2.3% [rural]). Furthermore, residents in northeast Haikou (Meilan District) had the highest prevalence of hypertension and diabetes mellitus (23.1% and 5.4%, respectively), while the elderly in the western part (Xiuying District) of Haikou had the highest prevalence of COPD and stroke (1.9% and 3.8%, respectively). The prevalence of hypertension and diabetes mellitus was the highest in the high-income (household monthly income≥6001 RMB) group (31.7% and 11.5%, respectively), whereas COPD and stroke were most prevalent in the low-income (household monthly income≤1500 RMB) group (1.3% and 2.1%, respectively). Similarly, highly educated elderly participants had the highest prevalence of hypertension and diabetes mellitus (29.4% and 12.1%, respectively) but the lowest prevalence of COPD (0.5%). Elderly manual workers had the lowest prevalence of hypertension and diabetes mellitus (19.4% and 4.3%, respectively), lower than in intellectual workers and retirees.

**Table 1:** Distribution of socio-demographic characteristics and risk factors for four chronic diseases.

Variables	Hypertension (N = 2449)		Diabetes mellitus (N = 749)		COPD (N = 95)		Stroke (N = 175)	
	Sample N*	Prevalence% (95%CI)^#^	Sample N	Prevalence% (95%CI)	Sample N	Prevalence% (95%CI)	Sample N	Prevalence% (95%CI)
**Gender**								
Male	1226	26.0(24.7, 27.3)	368	7.8(7.0, 8.6)	73	1.6(1.2, 2.0)	106	2.3(1.9, 2.7)
Female	1223	26.1(24.8, 27.4)	381	8.2(7.4, 9.0)	22	0.5(0.3, 0.7)	69	1.5(1.1, 1.9)
Total	2449	26.0(25.1, 26.9)	749	8.0(7.4, 8.5)	95	1.0(0.8, 1.2)	175	1.9(1.6, 2.2)
**Age group (years)**								
60–69	1052	20.5(19.4, 21.6)	363	7.1(6.4, 7.8)	39	0.8(0.5, 1.1)	68	1.3(1.0, 1.6)
70–79	917	31.9(30.2, 33.6)	285	10.0(8.9, 11.1)	28	1.0(0.6, 1.4)	66	2.3(1.8, 2.8)
≥80	480	34.1(31.6, 36.6)	101	7.2(5.8, 8.6)	28	2.0(1.3, 2.7)	41	2.9(2.0, 3.8)
**Residence**								
Urban	1620	31.2(29.9, 32.5)	570	11.0(10.1, 11.9)	33	0.6(0.4, 0.8)	77	1.5(1.2, 1.8)
Rural	829	19.7(18.5, 20.9)	179	4.3(3.7, 4.9)	62	1.5(1.1, 1.9)	98	2.3(1.8, 2.8)
**Region**								
West	476	23.1(21.3, 24.9)	112	5.4(4.4, 6.4)	40	1.9(1.3, 2.5)	78	3.8(3.0, 4.6)
Central	617	25.2(23.5, 26.9)	177	7.2(6.2, 8.2)	14	0.6(0.3, 0.9)	30	1.2(0.8, 1.6)
Mid-south	533	24.3(22.5, 26.1)	155	7.1(6.0, 8.2)	24	1.1(0.7, 1.5)	29	1.3(0.8, 1.8)
Northeast	823	30.4(28.7, 32.1)	305	11.3(10.1, 12.5)	17	0.6(0.3, 0.9)	38	1.4(1.0, 1.8)
**Monthly household income (RMB)**								
≤1500	572	21.7(20.2, 23.3)	152	5.8(4.9, 6.7)	35	1.3(0.9, 1.8)	56	2.1(1.6, 2.7)
1501–6000	1230	26.0(24.7, 27.2)	363	7.7(6.9, 8.5)	50	1.1(0.8, 1.4)	86	1.8(1.4, 2.2)
≥6001	647	31.7(29.7, 33.7)	234	11.5(10.1, 12.9)	10	0.5(0.2, 0.8)	33	1.6(1.1, 2.2)
**Education**								
Primary school or below	1097	25.1(23.8, 26.4)	270	6.2(5.5, 6.9)	49	1.1(0.8, 1.4)	97	2.2(1.7, 2.3)
High school	1013	26.1(24.7, 27.5)	339	8.8(7.9, 9.7)	40	1.0(0.7, 1.4)	57	1.5(1.0, 2.2)
Technical school or above	339	29.4(26.7, 32.0)	140	12.1(10.3, 14.0)	6	0.5(0.1, 0.9)	21	1.8(0.7, 2.7)
**Occupation**								
Intellectual worker	149	27.6(25.0, 32.8)	60	11.1(8.7, 14.1)	6	1.1(0.2, 2.0)	14	2.6(1.8, 2.7)
Manual worker	823	19.6(18.2, 20.6)	182	4.3(3.7, 4.9)	53	1.3(0.1, 1.6)	76	1.8(1.1, 1.9)
Retiree	1477	31.5(30.4, 33.0)	507	10.8(10.0, 11.8)	36	0.8(0.5, 1.1)	85	1.8(1.0, 2.6)
**Smoking status**								
Never	2022	26.3(25.3, 27.3)	639	8.3(7.7, 8.9)	57	0.7(0.5, 0.9)	124	1.6(1.3, 1.9)
Past smoker	280	21.2(19.0, 23.4)	65	5.0(3.8, 6.2)	15	1.1(0.5, 1.7)	31	2.4(1.6, 3.2)
Current smoker	147	38.0(33.1, 42.8)	45	11.7(8.5, 14.9)	23	5.9(3.5, 8.2)	20	5.2(3.0, 7.4)
**Alcohol intake**								
No	2159	26.6(25.6, 27.6)	670	8.3(7.7, 8.9)	83	1.0(0.8, 1.2)	162	2.0(1.7, 2.3)
Yes	290	22.6(20.3, 24.9)	79	6.2(4.9, 7.5)	12	0.9(0.4, 1.5)	13	1.0(0.5, 1.6)
**BMI (kg/m**^**2**^**)**								
<24	922	25.7(24.4, 27.2)	285	8.0(7.2, 9.0)	38	1.1(0.8, 1.4)	61	1.7(1.3, 2.1)
24≤BMI<28	375	38.1(34.9, 41.0)	140	14.2(12.1, 16.5)	4	0.4(0.0, 0.8)	15	1.5(0.7, 2.3)
≥28	96	37.8(32.0, 44.0)	38	15.0(12.0, 22.9)	5	2.0(0.3, 4.9)	12	4.7(2.4, 9.1)
Subtotal	1393	28.9(27.8, 30.4)	463	9.6(8.9, 10.7)	47	1.0(0.7, 1.3)	88	1.8(1.5, 2.3)

*Absolute numbers in the actual study sample.

^#^Respective row percentages and 95% confidence intervals.

Current smokers had the highest prevalence of hypertension (38.0%), diabetes mellitus (11.7%), COPD (5.9%), and stroke (5.2%), and alcohol abstainers had a slightly higher prevalence of these four chronic conditions than the elderly who consumed alcohol (hypertension 26.6%[alcohol abstainers] vs. 22.6%[alcohol consumer]; diabetes mellitus 8.3%[alcohol abstainers] vs. 6.2%[alcohol consumer]; COPD 1.0%[alcohol abstainers] vs. 0.9%[alcohol consumer]; stroke 2.0%[alcohol abstainers] vs. 1.0%[alcohol consumer]). Finally, participants with a BMI≥28 had the highest prevalence of the four chronic conditions (hypertension 43.2%, diabetes mellitus 17.5%, COPD 2.6%, stroke 5.8%) (Table 1). An additional data file shows this in more detail [see community elderly de-identified.sav]. In the binary logistic regression models (Table 2), male gender was strongly associated with COPD (OR_women vs. men_ = 0.33, 95%CI = 0.14–0.77). There were graded positive associations between age and the four chronic conditions (OR_70-79 years vs. 60–69 years, hypertension_ = 1.76, 95%CI = 1.52–2.03; OR_≥80 years vs. 60–69 years, hypertension_ = 2.02, 95%CI = 1.66–2.47; OR_70-79 years vs. 60–69 years, diabetes mellitus_ = 1.36, 95%CI = 1.09–1.69; OR_≥80 years vs. 60–69 years, diabetes mellitus_ = 1.46, 95%CI = 1.08–1.99; OR_≥80 years vs. 60–69 years, COPD_ = 2.87, 95%CI = 1.30–6.34; OR_≥80years vs. 60-69years, stroke_ = 1.85, 95%CI = 1.01–3.39). There were associations of residence with hypertension, diabetes mellitus and stroke, although northeast and mid-south residents had higher odds of hypertension and diabetes mellitus and residents in the west had the highest odds of COPD and stroke. Hypertension, COPD and stroke were associated with smoking status (OR_current smoker vs. never smoker, hypertension_ = 1.46, 95%CI = 1.09–1.96; OR_current smoker vs. never smoker, COPD_ = 4.39, 95%CI = 2.00–9.66; OR_current smoker vs. never smoker, stroke_ = 2.88, 95%CI = 1.40–5.91). Additionally, alcohol abstainers had higher odds of stroke (OR_consumer vs. abstainers_ = 0.32, 95%CI = 0.14–0.73), and chronic conditions were positively associated with BMI (OR_24≤BMI<28 vs. BMI<24, hypertension_ = 1.58, 95%CI = 1.35–1.85; OR_BMI≥28 vs. BMI<24, hypertension_ = 1.69, 95%CI = 1.28–2.21; OR_24≤BMI<28 vs. BMI<24, diabetes mellitus_ = 1.49, 95%CI = 1.19–1.86; OR_BMI≥28 vs. BMI<24, diabetes mellitus_ = 1.80, 95%CI = 1.24–2.62; OR_BMI≥28 vs. BMI<24, COPD_ = 2.76, 95%CI = 1.04–7.34; OR _BMI≥28 vs. BMI<24, stroke_ = 3.54, 95%CI = 1.84–6.82) (Table 2).

**Table 2:** Binary logistic regression analysis of factors associated with four chronic diseases.

Variables	Hypertension		Diabetes mellitus		COPD		Stroke	
	OR	95%CI	OR	95%CI	OR	95%CI	OR	95%CI
**Gender**								
Male	1.00	Reference	1.00	Reference	1.00	Reference	1.00	Reference
Female	0.99	0.86–1.15	1.04	0.83–1.29	0.33*	0.14–0.77	0.64	0.39–1.07
**Age group (years)**								
60–69	1	Reference	1.00	Reference	1.00	Reference	1.00	Reference
70–79	1.76***	1.52–2.03	1.36**	1.09–1.69	1.35	0.65–2.78	1.48	0.90–2.43
≥80	2.02***	1.66–2.47	1.46*	1.08–1.99	2.87**	1.30–6.34	1.85*	1.01–3.39
**Residence**								
Urban	1.00	Reference	1.00	Reference	1.00	Reference	1.00	Reference
Rural	0.72***	0.60–0.86	0.57***	0.42–0.78	1.20	0.48–2.99	2.80**	1.51–5.20
**Region**								
West	1.00	Reference	1.00	Reference	1.00	Reference	1.00	Reference
Central	1.05	0.85–1.29	1.07	0.76–1.50	0.55	0.24–1.26	0.28**	0.13–0.60
Mid-south	1.31**	1.09–1.59	1.45*	1.04–2.01	0.17***	0.07–0.43	0.39**	0.22–0.69
Northeast	1.32**	1.09–1.60	1.81***	1.34–2.45	0.35*	0.15–0.83	0.38**	0.21–0.68
**Monthly household income (RMB)**								
≤1500	1.00	Reference	1.00	Reference	1.00	Reference	1.00	Reference
1501~6000	1.08	0.91–1.28	1.25	0.98–1.59	0.79	0.41–1.54	1.27	0.76–2.11
≥6001	1.15	0.93–1.43	1.15	0.84–1.58	0.41	0.12–1.40	1.20	0.55–2.62
**Education**								
Middle school or below	1.00	Reference	1.00	Reference	1.00	Reference	1.00	Reference
High school	0.91	0.78–1.05	1.25	0.98–1.59	2.29*	1.12–4.71	0.75	0.44–1.26
Junior college and university	0.82	0.66–1.02	1.15	0.84–1.58	1.76	0.53–5.83	1.13	0.54–2.37
**Occupation**								
Intellectual worker	1.00	Reference	1.00	Reference	1.00	Reference	1.00	Reference
Manual worker	0.77	0.57–1.02	0.67	0.43–1.04	1.07	0.32–3.58	0.49	0.20–1.20
Retiree	1.06	0.82–1.37	1.13	0.78–1.62	0.49	0.16–1.57	0.83	0.36–1.93
**Smoking status**								
Never	1.00	Reference	1.00	Reference	1.00	Reference	1.00	Reference
Past smoker	0.78*	0.63–0.98	0.76	0.52–1.09	0.76	0.32–1.81	1.21	0.63–2.33
Current smoker	1.46*	1.09–1.96	1.41	0.93–2.16	4.39***	2.00–9.66	2.88**	1.40–5.91
**Alcohol intake**								
No	1.00	Reference	1.00	Reference	1.00	Reference	1.00	Reference
Yes	0.93	0.75–1.14	0.90	0.64–1.25	0.73	0.33–1.62	0.32**	0.14–0.73
**BMI (kg/m**^**2**^**)**								
<24	1.00	Reference	1.00	Reference	1.00	Reference	1.00	Reference
24≤BMI<28	1.58***	1.35–1.85	1.49**	1.19–1.86	0.46	0.16–1.32	1.15	0.63–2.08
≥28	1.69***	1.28–2.21	1.80**	1.24–2.62	2.76*	1.04–7.34	3.54***	1.84–6.82

Odds ratios (ORs: specific chronic disease vs. no specific chronic disease) and 95% confidence intervals (95%CIs) from binary logistic regression.

*p<0.05

**p<0.01

***p<0.001

## Discussion

Previous studies reported that both urban and rural areas in Haikou showed a lower prevalence of chronic conditions than some of the northern provinces of China [12–15] and other developing countries [16–19]. According to previous finding, the lower prevalence of chronic diseases in Haikou could be ascribed to there being fewer smokers (18.3%), lower alcohol consumption (19.7%) and fewer overweight (17.5%) and obese (4.1%) people [20]. In the current study, near one third of the sample reported at least one chronic condition implies the severity of chronic diseases among the Haikou older adults. Among the four chronic diseases, hypertension was most commonly reported chronic condition. The prevalence of hypertension in our study sample is similar with findings from the 2007–08 China National Diabetes and Metabolic Disordered Study that indicated 26.6% of adults aged 20 years and above has hypertension [21]. It was reported that cardiovascular and cerebrovascular diseases are the leading cause of death in China and that hypertension is its most common preventable risk factor [22]. Despite the high prevalence of hypertension among the Chinese communities, numerous studies revealed that low rate of hypertension treatment and control were due to lack of awareness [23, 24]. As such, extensive health education on hypertension couple with periodic physical examination should be enhanced among the elderly in Haikou.

Of note, the prevalence of self-reported stroke in this study (1.9%) is relatively higher than the most recently reported in a nationally-represented study conducted collectively in 31 provinces in China, where the survey reported an age-standardized prevalence of stroke of 6.17 per 1000 [25]. The stroke burden in China has reported to increase over the last 30 years [25]. Given the already immense and fast-increasing burden of stroke, health policy should develop and implement an emergency action plan addressing the primary prevention, possibly include strategies to tackle unhealthy behaviors that increase the risk of stroke.

In regards to COPD, similar to previously reported [26], COPD was more prevalent in males and in current smokers. The fact that COPD is being more prevalent in males could be attributed to the fact that smoking is more common among the elderly males. The rate of cigarette smoking is extremely high in China. The estimated smoking rate in men is 67% and in women is 4%. WHO estimates there are close to 300 million Chinese smokers, which is more than the entire US population and, collectively, they consume an estimated 1.7 trillion cigarettes per year [27]. Previous study revealed that elderly who experience more health problems and psychological distress are more likely to try to stop smoking than those with lower health and psychological distress [28], of which the latter group may need further motivational and educational strategies to support smoking cessation than the former [29]. As China’s elderly population is projected to continue increase rapidly, the health, social, and economic costs of smoking will continue to rise among the smoker. Therefore, tobacco cessation initiatives are important on disease and mortality risk among the elderly.

The current study also found that the prevalence of all four chronic diseases increased with age, likewise found in other studies [30, 31]. As such young adults and middle age should be made known of the alarming trend and statistic in order for them to consider healthier lifestyles to achieve healthy aging. Previous study reported that cardiovascular disease was the main, chronic disease among intellectual compared to manual workers [32]. Nonetheless, this study did not show that intellectual workers presented significantly different prevalence of stroke compared to manual workers. The unobvious labor intense difference among elderly could be the reason. Still, findings suggest wellness program in community to increase active lifestyles should be encouraged.

Further, this study also found rural residence was associated with a significantly lower prevalence of hypertension and diabetes mellitus but a higher prevalence of stroke. The findings are consistent with previous study and can be explained by the delayed engagement in health risk behaviours occurring as a result of disparities in geography, environment and health service availability [33, 34]. In this study, alcohol consumers had lower odds of stroke, and this finding was inconsistent with the results of previous research [35], and the associations between alcohol consumption with hypertension, diabetes mellitus and COPD were not significant in this study. Further studies will be needed to confirm these associations.

Body mass index (BMI) has routinely been considered a risk factor for chronic conditions [31, 33, 34]. Although the prevalence of being overweight or obese individuals in Haikou was lower than the average in China [36], overweight and obesity have increasing become a major public health problem nationwide in China. Rapid economic growth in China has led to changes in dietary and physical activities, as such prevention and control of overweight and obesity is of great urgency. Further, the results of this study also showed that chronic conditions vary significantly across the four regions of Haikou. This is perhaps explained by the fact that the Eastern region has developed faster than other parts of Haikou, leading to changes in mental stress, health risk behaviours and dietary habits.

Although there were evidence of socio-economic disparities in health in China [37], our findings concur with previous findings that reported lack of strong evidence for the association of chronic conditions with income and education level [38, 39]. The socio-economic disparities in chronic conditions in China are not completely consistent with those in developed countries. Further large scale studies are warranted to reach a definite conclusion.

This research has some limitations, including potential errors in self-reporting of chronic conditions, participant recall bias, and interviewer bias. The major potential limitation of this study is the underreporting of chronic diseases among the participants. Although we tried to verify the prevalence of chronic diseases among our participants through their health records, the health information could not be updated in time; this was due, probably, to the low rate of regular physical examination among older adults, especially in rural area. In light of this, we adopted the precautions of using questions such as “have you been diagnosed with hypertension?” rather than “Are you a hypertensive patient?”, and referring throughout the article to “self-reported prevalence”. The second limitation of this study is the cross-sectional study design, which limits our ability to establish causal relationships. Further, the analyses in this manuscript is limited to single morbidity, it would be beneficial to for future study that take into consideration of studying the correlates of multi-comorbidities as it is common among the elderly.

In conclusion, despite the potential limitations, this study provides important insight into the self-reported prevalence of multiple chronic conditions and their related risk factors in the elderly population of Haikou. Elderly people in Haikou are exposed to health-harming behaviours and obesity, increasing their risks of chronic conditions. This baseline evidence may be helpful for identifying the health priorities for people in Haikou; this knowledge could provide practical guidance for developing relevant health policies. However, the prevalence of chronic diseases and the associated factors in the target population should continue to be monitored in the long term.

## Supporting information

S1 Dataset Community elderly de-identified. (SAV)

## Abbreviations

COPD: chronic obstructive pulmonary disease
BMI: body mass index
CI: confidence interval
